# Modeling *Staphylococcus epidermidis*-Induced Non-Unions: Subclinical and Clinical Evidence in Rats

**DOI:** 10.1371/journal.pone.0147447

**Published:** 2016-01-21

**Authors:** Arianna Barbara Lovati, Carlo Luca Romanò, Marta Bottagisio, Lorenzo Monti, Elena De Vecchi, Sara Previdi, Riccardo Accetta, Lorenzo Drago

**Affiliations:** 1 Cell and Tissue Engineering Laboratory, IRCCS Galeazzi Orthopaedic Institute, Milan, Italy; 2 Dipartimento di Chirurgia Ricostruttiva e delle Infezioni Osteo-articolari, IRCCS Galeazzi Orthopaedic Institute, Milan, Italy; 3 Orthopaedics and Traumatology, IRCCS Galeazzi Orthopaedic Institute, Milan, Italy; 4 Laboratory of Clinical Chemistry and Microbiology, IRCCS Galeazzi Orthopaedic Institute, Milan, Italy; 5 Laboratory of Cancer Cachexia AIRC Start-Up, Oncology Department, Mario Negri Institute for Pharmacological Research, Milan, Italy; 6 Department of Biomedical Science for Health, University of Milan, Milan, Italy; 7 Department of Veterinary Science and Public Health, University of Milan, Milan, Italy; Harvard Medical School, UNITED STATES

## Abstract

*S*. *epidermidis* is one of the leading causes of orthopaedic infections associated with biofilm formation on implant devices. Open fractures are at risk of *S*. *epidermidis* transcutaneous contamination leading to higher non-union development compared to closed fractures. Although the role of infection in delaying fracture healing is well recognized, no *in vivo* models investigated the impact of subclinical low-grade infections on bone repair and non-union. We hypothesized that the non-union rate is directly related to the load of this commonly retrieved pathogen and that a low-grade contamination delays the fracture healing without clinically detectable infection. Rat femurs were osteotomized and stabilized with plates. Fractures were infected with a characterized clinical-derived methicillin-resistant *S*. *epidermidis* (10^3^, 10^5^, 10^8^ colony forming units) and compared to uninfected controls. After 56 days, bone healing and osteomyelitis were clinically assessed and further evaluated by micro-CT, microbiological and histological analyses. The biofilm formation was visualized by scanning electron microscopy. The control group showed no signs of infection and a complete bone healing. The 10^3^ group displayed variable response to infection with a 67% of altered bone healing and positive bacterial cultures, despite no clinical signs of infection present. The 10^5^ and 10^8^ groups showed severe signs of osteomyelitis and a non-union rate of 83–100%, respectively. The cortical bone reaction related to the periosteal elevation in the control group and the metal scattering detected by micro-CT represented limitations of this study. Our model showed that an intra-operative low-grade *S*. *epidermidis* contamination might prevent the bone healing, even in the absence of infectious signs. Our findings also pointed out a dose-dependent effect between the *S*. *epidermidis* inoculum and non-union rate. This pilot study identifies a relevant preclinical model to assess the role of subclinical infections in orthopaedic and trauma surgery and to test specifically designed diagnostic, prevention and therapeutic strategies.

## Introduction

Fracture non-unions represent a great clinic and surgical challenge, in particular when associated with bacterial infections. Septic delayed- or non-union fractures have limited and often difficult treatment options, requiring prolonged hospitalization and antibiotic therapy, with a high socioeconomic impact [1, 2].

Although the development of a fracture non-union depends on many factors, including the type and site of fracture, the treatment and host response, open fractures are definitely at higher risk of non-union—5% to 100%, depending on the degree of exposure and contamination [3]—than those undergoing osteosynthesis for closed, not contaminated fractures (1–2%) [4, 5].

Although this observation points out the direct relationship between fracture contamination, infection and non-union development, we currently have no animal models or data from human studies concerning the impact of subclinical, low-grade infections on bone healing and non-union. Sporadic clinical observations showed that low-virulent pathogens, like certain coagulase-negative staphylococci, might be related to non-unions or pain at the fracture site even in the absence of clinical signs of infection [6].

*Staphylococcus aureus* and *Staphylococcus epidermidis* are the most common pathogens involved in orthopaedic infections and account for 70–90% of the cases after elective surgery [7].

*S*. *epidermidis* is a harmless commensal inhabitant of human skin lacking the capability to penetrate the host [8]. Therefore, the *S*. *epidermidis*-related infection is caused by its delivery from the skin to the host tissues in case of open fractures and surgical procedures [9].

*S*. *epidermidis* is also one of the leading causes of infections associated with biofilm formation because of its high ability to adhere and colonize implant medical devices, such as catheters, heart valves and orthopaedic prosthesis [10–12].

The biofilm formation in *S*. *epidermidis* orthopaedic infections makes less effective the antimicrobial treatment and increases the emergence of antibiotic-resistant strains such as methicillin-resistant *S*. *epidermidis* (MRSE), a common osteomyelitis-inducing pathogen [13].

Concerning the consequence of *S*. *epidermidis*-associated infections in orthopaedic implants, animal models are mandatory to investigate the pathogenesis of non-union-related infections, with particular reference to subclinical infections. To our knowledge, there are many rodent models of osteomyelitis and septic arthritis, but only few studies did investigate the fracture repair in the presence of *S*. *aureus* infection by performing critical defects in long bones [1, 14–17], while no modeling of *S*. *epidermidis* implant-related infection in osteosynthesis has been described so far.

Specifically, a few studies did investigate clinical MRSE strains in developing intravascular or urinary tract infections [18–20] as well as in wound healing and in subcutaneous models [9, 21]. Animal models of prosthetic joint and medullary canal infections have also been studied associating a clinical MRSE strain to stainless steel implants [22–24]. To the best of our knowledge, there is not a suitable animal model to mimic MRSE-induced non-union fractures. Moreover, there are no data concerning the effect of low bacteria inocula on fracture healing after osteosynthesis. Our hypothesis is that the rate of non-union, induced by a commonly isolated pathogen, like *S*. *epidermidis*, is directly related to the inoculated bacterial load and that very low bacterial inocula may be associated with a higher rate of non-union development compared to uninfected controls, despite the absence of local or general signs or symptoms of a post-surgical infection.

For the first time, we propose a rat model of dose-dependent MRSE-induced non-union synthesized with metal implants both to demonstrate the role of subclinical infections in impairing the fracture healing and to create a model useful to investigate novel treatments for non-unions.

## Materials and Methods

### Ethics Statement

The whole study was approved by the Mario Negri Institute for Pharmacological Research (IRFMN) Animal Care and Use Committee (IACUC) (Permit N. 06/2014-PR). The animals were housed at the Institute's Animal Care Facilities that meet international standards. The IRFMN adheres to the principles set out in the following laws, regulations, and policies governing the care and use of laboratory animals: Italian Governing Law (D.lgs 26/2014; Authorization n.19/2008-A issued March 6, 2008 by Ministry of Health); Mario Negri Institutional Regulations and Policies providing internal authorization for persons conducting animal experiments (Quality Management System Certificate–UNI EN ISO 9001:2008 –Reg. N° 6121); the NIH Guide for the Care and Use of Laboratory Animals (2011 edition) and EU directives and guidelines (EEC Council Directive 2010/63/UE). The Statement of Compliance (Assurance) with the Public Health Service (PHS) Policy on Human Care and Use of Laboratory Animals has been recently reviewed (9/9/2014) and will expire on September 30, 2019 (Animal Welfare Assurance #A5023-01). The animals were regularly checked by a certified veterinarian responsible for health monitoring, animal welfare supervision, experimental protocols and procedure revision. All surgeries were performed under general anesthesia, and all efforts were made to minimize suffering.

### Study design

Twenty-four 12-weeks-old Wistar male rats weighing 300–350 g (Harlan, Italy) were included in this study. The rats were randomly assigned to one of the experimental groups (n = 6 each group): the control group (CTRL) was injected with 30 μl phosphate-buffered saline (PBS); the 10^3^ MRSE, 10^5^ MRSE, and 10^8^ MRSE groups were injected with 30 μl of a bacterial suspension containing 10^3^, 10^5^, and 10^8^ colony forming units (CFU) of MRSE, respectively.

After 8 weeks, micro-CT scanning, microbiological and histological analyses were performed to assess the bone healing and infection, and Scanning Electron Microscopy (SEM) was carried out to visualize the biofilm formation.

### Bacterial strain characterization

The MRSE strain GOI1153754-03-14 used in this study was isolated at the Laboratory of Clinical Chemistry and Microbiology (IRCCS Galeazzi Orthopaedic Institute, Milan, Italy). The strain derived from infected knee prosthesis of a patient undergone implant revision at the Center for Reconstructive Surgery of Osteoarticular Infections (IRCCS Galeazzi Orthopaedic Institute, Milan, Italy). This strain was selected for its antibiotic susceptibility pattern and its capacity to produce biofilm on prosthetic materials *in vitro*.

### Identification and antibiotic susceptibility testing

Microbiological identification was performed at a phenotypic and genotypic level. Phenotypic identification carried out on VITEK2 System (Biomerieux, France) was subsequently confirmed by pyrosequencing analysis (PSQ96RA, Diatech, Italy) of DNA of variable regions V1 and V3 of the 16S rRNA gene by using primers reported in a previous study [25]. Amplified sequences (about 60–80 bp) were compared with sequences available in BLAST search. Antimicrobial susceptibility testing and determination of the minimum inhibitory concentration (MIC) were carried out on Vitek2 System using the card AST 632 specific for staphylococci.

### Biofilm formation assay by crystal violet staining

Biofilm production was evaluated according to spectrophotometric assay [26] that was repeated in triplicate. Briefly, the MRSE strain was grown on blood agar plates. After overnight incubation at 37°C in aerobiosis, a 0.5 McFarland suspension was prepared and 20μl aliquots were inoculated in 96-well plates containing 180μl of Tryptose Soy Broth (TSB; BioMérieux). After an overnight incubation at 37°C in aerobiosis, the medium was refreshed and plates were incubated for a further 48 hours at 37°C. Un-inoculated wells containing only TSB were used as negative control. At the end of incubation, the wells were washed with PBS (Gibco, Italy) to remove bacteria not included in biofilm. Once dried, wells were stained with 200μl of 5% crystal violet solution (Merck, Germany) for 10 minutes, and then washed. After air-drying, 200μl of absolute ethanol were added to solubilize the dye attached to the biofilm. The optical density (OD) of each well was measured at 595 nm by using a microplate reader (Multiskan FC, Thermo Scientific, Italy). Strains were classified as strong, moderate or weak producers of biofilm, according to criteria defined by Stepanovic et al [27], which are based on the comparison between the OD of strain under testing and a cut-off value (ODc) defined as three standard deviation above the mean OD of the negative control (un-inoculated broth). In particular, if the sample OD (ODs) is less than the ODc, the strain is considered as a non-biofilm producer. If the ODs value is comprised between 1 and 2 ODc, the strain is classified as a weak producer. If the ODs values is comprised between 2 and 4 ODc, the strain is classified as a moderate producer. Finally, if the ODs value is greater than 4 ODc, the strain is classified as a strong producer.

### Preparation of MRSE for inoculation into the femur fracture

Previously characterized MRSE strain was cultured onto Mannitol Salt Agar (BioMérieux) at 37°C overnight. To prepare the inoculum, a selected colony was cultured into Brain Heart Infusion Broth (BHI, BioMérieux) and incubated for 16 hours at 37°C. The bacterial suspension was washed twice and the obtained pellet was suspended in sterile saline to obtain a 10 McFarland turbidity equal to about 3×10^9^ CFU/ml. The bacterial suspension was then serially diluted with sterile saline solution to obtain the desired bacterial load of 1x10^3^, 1x10^5^ and 1x10^8^ CFU/30μl. Bacterial inocula were verified and confirmed by agar plate counting procedures. The bacterial suspension was used within 2 hours and stored at 4°C until use.

### *In vivo* surgical procedures

Twenty-four 12-weeks old male Wistar rats (mean body weight 337.6±10.2 g) were used for the experiments. The rats were maintained in controlled conditions of temperature and lightning and fed with autoclaved food and water provided *ad libitum*. All pre-surgical and surgical procedures on the animals were performed under a laminar flow hood.

**Fig 1:**
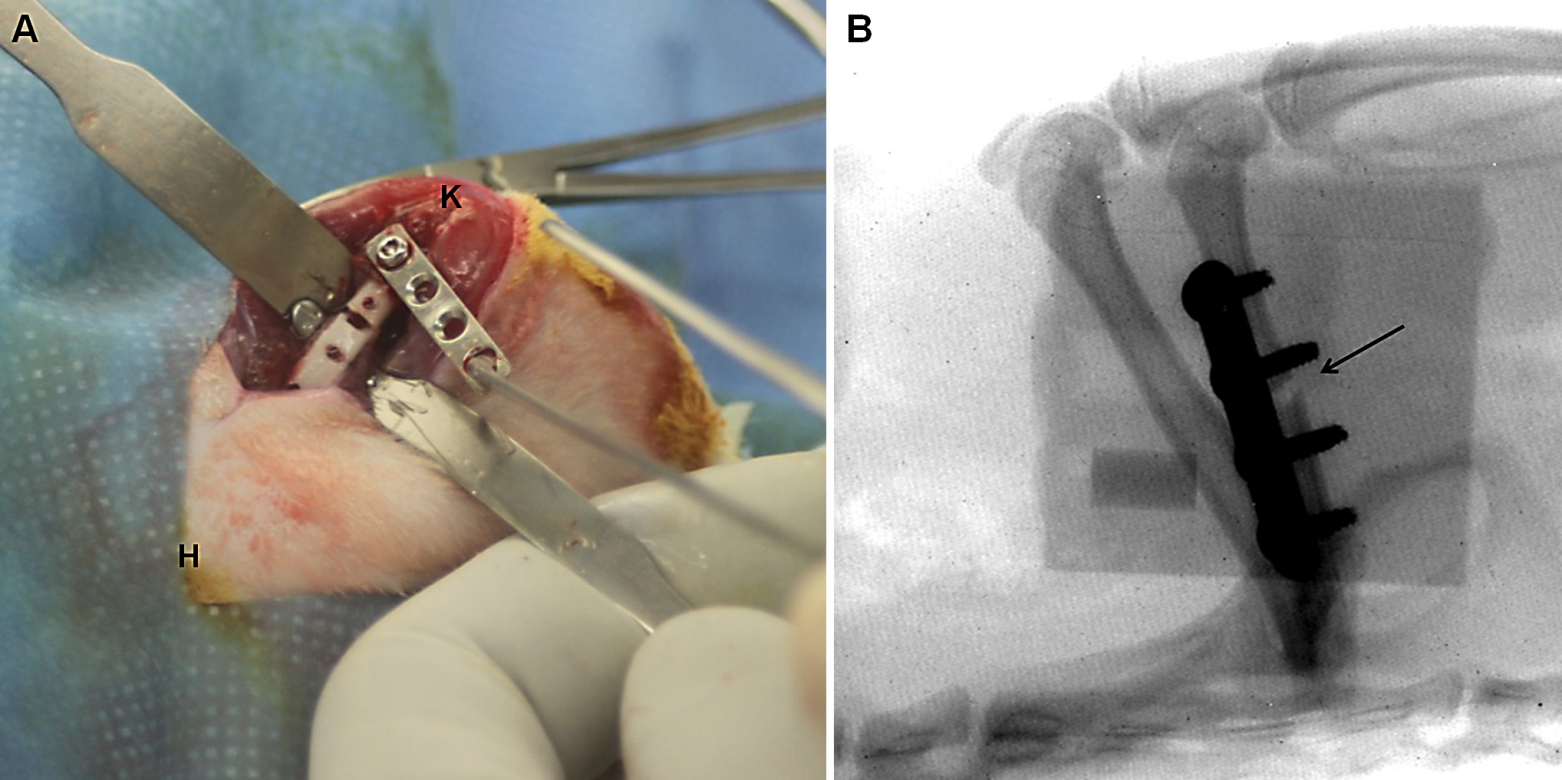
Plate positioning and postoperative analysis. (A) Plate diversion from the femur and the creation of a 1 mm non-critical midshaft full-thickness defect. The anatomical sites are reported as knee (K) and hip (H) joints. (B) Fluoroscopic examination of the femoral fracture and the correct plate position. The fracture of the femoral midshaft is shown by a black arrow.

The rats were anesthetized via inhalation of isoflurane (3%; Merial, Italy) and maintained with an intraperitoneal injection of ketamine hydrochloride (80mg/kg; Imalgene, Merial) and medetomidine hydrochloride (1mg/kg; Domitor, Pfizer, Italy). All animals received a preoperative intramuscular single injection of cefazolin (30mg/kg; Cefamezin, Teva, Italy) and a subcutaneous treatment with carprofen (5mg/kg; Rimadyl, Pfizer). Using aseptic technique, all rats were submitted to a midshaft osteotomy of the right femur. Briefly, after shaving and disinfection, a longitudinal 2.5 cm skin incision was performed through a lateral approach to expose the femoral shaft by blunt dissection between the lateral *vastus* muscle and the femoral biceps muscle. Using the distal screw as a pivot, the plate was diverted from the femur and a 1 mm non-critical midshaft full-thickness defect was created after a localized periosteal elevation with an electric circular saw under continuous sterile saline irrigation (Fig 1A). A compression stainless steel four-hole-mini-plate (length 20mm, width 4mm, height 1mm) (Mini Fragment plate) was fixed on the anterolateral surface of the femoral diaphysis using four 1.5mm Ø bicortical screws (all from Zimmer®, Germany).

In the infected groups, a volume of 30μl of the bacterial suspension, corresponding to an inoculum of about 1x10^3^, 1x10^5^ and 1x10^8^ CFU/rat was injected into the femoral defect and the suspension was allowed to spread throughout the medullary canal. The sham-inoculated control group received an inoculum of 30μl sterile PBS. Then the muscular planes were closed with a continuous suture with Vycril 4/0 and the skin with separated stitches with Prolene 4/0 (Johnson&Johnson, Italy). The stability of the fracture was firstly manually assessed, then confirmed by fluoroscopic examination (Fig 1B). Atipamezole (1mg/kg; Antisedan, Pfizer) was administered subcutaneously to recover the animals from general anesthesia. The animals were then housed in separate cages under an infrared lamp and monitored until the effects of anesthesia had worn off. About 24 hours later, animals were couple caged, monitored daily for general status and welfare, clinical signs of infection, lameness, weight bearing, swelling, local hyperemia, wound healing, serous exudate, hematoma, pain and suffering. The pain was controlled with buprenorphine (0.1mg/kg SC; Temgesic, Schering Plough, Italy) immediately after surgery.

After 8 weeks, the rats were euthanized by CO_2_ inhalation to perform the investigations.

Anatomical dissection was performed under a laminar flow hood and in sterile conditions; the soft tissues were inspected for gross appearance, then stripped off the cortical bone surface, and the femurs were aseptically retrieved.

### Animal weight and blood analyses

The body weight was measured in all animals at day 0 (day of operation) and weekly until the explantation. As an indicator of infection, hematology for each rat was performed on blood samples. Blood samples were collected from the tail vein on day 0 and day 14, and directly from the left ventricle immediately after sacrifice (day 56) to determine the peripheral neutrophil count, acting as first defenders against infections (n = 6 per group). The blood was promptly transferred to centrifuge tubes containing the appropriate anticoagulants for cell counting. Blood samples in 0.5M EDTA (Sigma Aldrich, Italy) were processed with an automatic cell counter (Sysmex XT-1800, Dasit, Italy) to obtain the white blood count and leukocyte formula.

### Micro-CT imaging and data analysis

Micro-CT imaging analysis was performed with an Explore Locus micro-CT scanner (GE Healthcare, Canada), without contrast agents. Immediately after sacrifice, the femurs (n = 6 per group) were removed, placed into a culture dish and scanned *en bloc*.

A micro-CT lower-resolution (Bin-2) protocol was performed using 80kV voltage, 400μA current with 400 msec exposure time per projection and 720 projections over 360° for a total scan time of approximately 24 minutes. The isotropic resolution of this protocol is 45μm. The 3D reconstructed images were viewed and analyzed using the MicroView software (version 2.1.2; GE Healthcare). Individual micro-CT images were qualitatively scored for osteomyelitis by two blinded observers according to the Odekerken’s grading scale [28]: 0, no abnormalities; 1, mild periosteal reaction, cortical thickening; 2, evident periosteal reaction, cortical thickening, and mild osteolysis; 3, extensive cortical thickening, cortical focal loss, evident osteolysis; 4, extensive cortical thickening, osteolysis, loss of cortical morphology. Bony bridging > 75% of the fracture gap was considered as healed fractures and bridging < 75% was seen as non-union fracture, according to others [1]. Furthermore, a histogram-based isosurface rendering was performed on the fracture region. Then, after scan calibration, using a phantom made of an epoxy-based resin that mimics hydroxyapatite and contains water and air inclusion, a cylindrical volume of interest (VOI, 780 mm^3^) including the fracture site was designed between the proximal and distal screw to quantitatively measure the bone formation. The bone volume (BV, mm^3^) and tissue mineral density (TMD, mg/cc) were measured within the identified VOI as highest sensitive parameters in early stage of fracture healing [29].

### Microbiological analysis

After 56 days, bacteria were recovered from samples (n = 5 per group) consisting in the plate, screws and peri-implant tissue. Samples were weighed and treated with dithiothreitol (DTT) to detach bacteria from the biofilm, as previously described [30]. Briefly, samples were immersed in a 0.1% w/v DTT (Sigma) solution in PBS and mechanically stirred for 15 min at RT. Samples were centrifuged and pellets suspended in 1 ml of the DTT eluate, 0.1 mL was then plated onto blood agar plates and inoculated in BHI broth. Plates were incubated for 48 hours at 37°C while incubation of broths at 37°C was prolonged for 15 days. Broths were daily checked for microbial growth. Then, 10μL from positive broths were plated onto blood agar plates which were incubated for 24 at 37°C. Gram-positive stained colonies were assessed for catalase test and for growth on Mannitol salt agar. Mannitol negative, coagulase negative, white, smooth, not hemolytic colonies on blood agar, resembling *S*. *epidermidis* were identified by pyrosequencing and counted. The (Log CFU)/g explant was determined by dividing the CFU number by the initial total weight of the sample. The limit of detection was set at 1 (Log CFU)/g. Phenotypic characteristics (biochemical and antibiotic susceptibility profiles) and sequences obtained by pyrosequencing were also compared with that of the *in vivo* injected *S*. *epidermidis* to confirm strain identity.

### Histological analysis

Femoral specimens (n = 5 per group) were fixed in 10% formalin for 24 hours. The bones were decalcified in Osteodec (Bio-Optica, Italy) for 7 days and dehydrated in alcohol scale before embedding the specimens in paraffin and cutting into 5 μm longitudinal sections. The slides were stained with haematoxylin and eosin (H&E) to assess morphology and with Gram staining for bacterial examination. Photomicrographs were captured using an Olympus IX71 light microscope and an Olympus XC10 camera (Japan). The samples were evaluated by two blinded observers to assess the percentage of the fracture healing and signs of osteomyelitis according to a grading score proposed in our previous study [31]. The Gram positive staining was evaluated as present or absent.

### Scanning Electron Microscopy analysis

After explantation, one sample per group was fixed in 2.5% paraformaldehyde and 2.5% glutaraldehyde in 0.1M Na-Cacodylate buffer (pH 7.4; all from Sigma) for 24 hours. After fixation, the samples were fixed for 1 hour in 1% osmium tetroxide (Sigma) in 0.1M Cacodylate buffer, then prepared to expose the plate surfaces and dehydrated in ethanol scale, mounted on aluminum stubs and sputter-coated with gold using a SEMPREP 2 Sputter Coater (Nanotech Ltd, UK). Observations were performed with a LEO 1400 EVO Scanning Electron Microscope (Zeiss, Germany) mixing secondary and backscattered electrons detectors. Images were acquired at 10kV at a working distance of 7 mm.

### Statistical analysis

The normal distribution of data was ascertained with the Shapiro-Wilk test. Comparisons among groups and time points were analyzed with two-way analysis of variance (ANOVA) (GraphPad Prism v5.00 Software, USA) coupled with Bonferroni’s post hoc test. Comparisons among groups were analyzed with one-way ANOVA corrected with Dunnett’s post hoc test. The interrater reliability of the examiners’ scores for micro-CT and histology were calculated with intraclass correlation coefficient (ICC): ICC = 1, perfect reliability; ICC > 0.75, excellent reliability. All data are expressed as means ± standard error (SE), unless specified otherwise. Values of *P*<0.05 were considered statistically significant.

## Results

### *In vitro* antibiotic susceptibility and biofilm production

The strain of *S*. *epidermidis* used in our infected non-union model was susceptible to gentamicin, vancomycin and fusidic acid (MIC ≤ 0.5μg/ml), erythromycin and daptomycin (MIC ≤ 0.25μg/ml), clindamycin and tigecycline (MIC ≤ 0.12μg/ml), linezolid and tetracycline (MIC ≤ 1μg/ml), teicoplanin (MIC = 4μg/ml), trimethoprim/sulfamethoxazole (MIC ≤ 10μg/ml). The strain was resistant to benzylpenicillin (MIC ≥ 0.5μg/ml), oxacillin, cefazolin, rifampicin and levofloxacin (MIC ≥ 4μg/ml). Based on the OD value (ODs vs ODc ratio: 6.38), the isolated strain was classified as a strong biofilm producer.

### Clinical examination

During the follow-up period, none of the animals included in any group died or had clinical evidence of implant or systemic infection, such as local signs of peri-implant inflammation (hyperemia or exudation), diarrhea and behavioral alterations.

**Fig 2:**
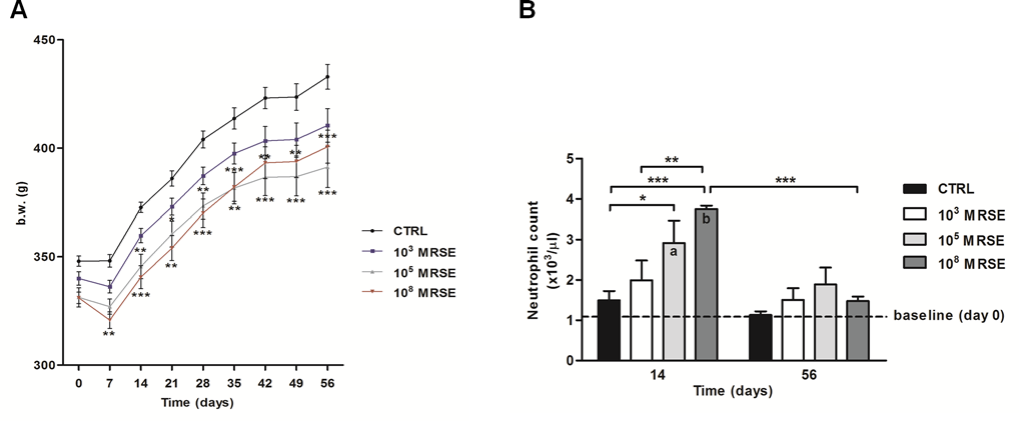
Clinical data. (A) The graph shows the numerical values of body weight (g) in the experimental groups over time. (B) The histogram shows the neutrophil count among experimental groups at day 14 and 56 after surgery. The dotted line represents the baseline at day 0 before surgery. Comparisons between groups and time points were analyzed with two-way ANOVA and Bonferroni’s post-hoc. Statistical significance was *P*<0.05 (*, a, b), *P*<0.01 (**) and *P*<0.001 (***), n = 6.

At day 0 and weekly, the body weight (b.w.) was determined and reported as numerical data for all groups (Fig 2A). During the acute phase (7 days after surgery), infection induced anorexia associated with a b.w. loss in all infected groups, particularly in the 10^8^ MRSE group compared to the control group (*P*<0.01). At the same time point, no b.w. loss occurred in the control group. Thereafter, in the control group, b.w. recovered progressively consistent with the standard Wistar rat-growing curve from day 14 until the end of the experiment. On the contrary, infected animals showed a less marked b.w. recovery during the same period; in particular, infected rats of the 10^8^ MRSE group recovered less b.w. than other infected groups from day 14 to day 35 after infection. A significant difference was found in the 10^8^ MRSE group compared to the controls at different time points (*P*<0.001 at 14, 28 and 56 days; *P*<0.01 at 21, 35, 42 and 49 days). Unexpectedly, after 35 days, the 10^8^ MRSE group showed a sensible b.w. regain reaching values similar to the 10^3^ MRSE group. Conversely, the 10^5^ MRSE group showed a permanent slow recovery of b.w. compared to other infected groups as well as to the control group with a significant difference at different time points (*P*<0.0001 at 35, 42, 49 and 56 days; *P*<0.01 at 14 and 28 days; *P<*0.05 at 21 days).

### Blood laboratory tests

The peripheral neutrophil count is reported as number of neutrophils x10^3^/μl compared to the baseline (day of surgery, day 0) at 14 and 56 days after surgery (Fig 2B).

Two weeks after infection, all animals exhibited an increase of the neutrophil count with respect to the basal values. In particular, both the 10^5^ and 10^8^ MRSE groups showed a significant increase of neutrophils compared to the basal values (*P*<0.01 and *P*<0.001, respectively) and compared to the control group (*P*<0.05 and *P*<0.001, respectively). A significant difference in neutrophil count was also found in the 10^8^ MRSE group compared with the 10^3^ MRSE group at two-weeks post-operatively (*P*<0.01). Interestingly, the post-operative neutrophil count of the 10^3^ MRSE group did not significantly differ from both the pre-operative basal values and from the control group at any time. In all groups, the peripheral neutrophil count almost normalized after eight weeks without showing any significant differences compared to the baseline. Interestingly, the 10^8^ MRSE group showed the most significant decrease also compared to the same group at 2 weeks (*P*<0.001). No significant differences were detected among the experimental groups at 8 weeks.

### Imaging diagnosis

**Fig 3:**
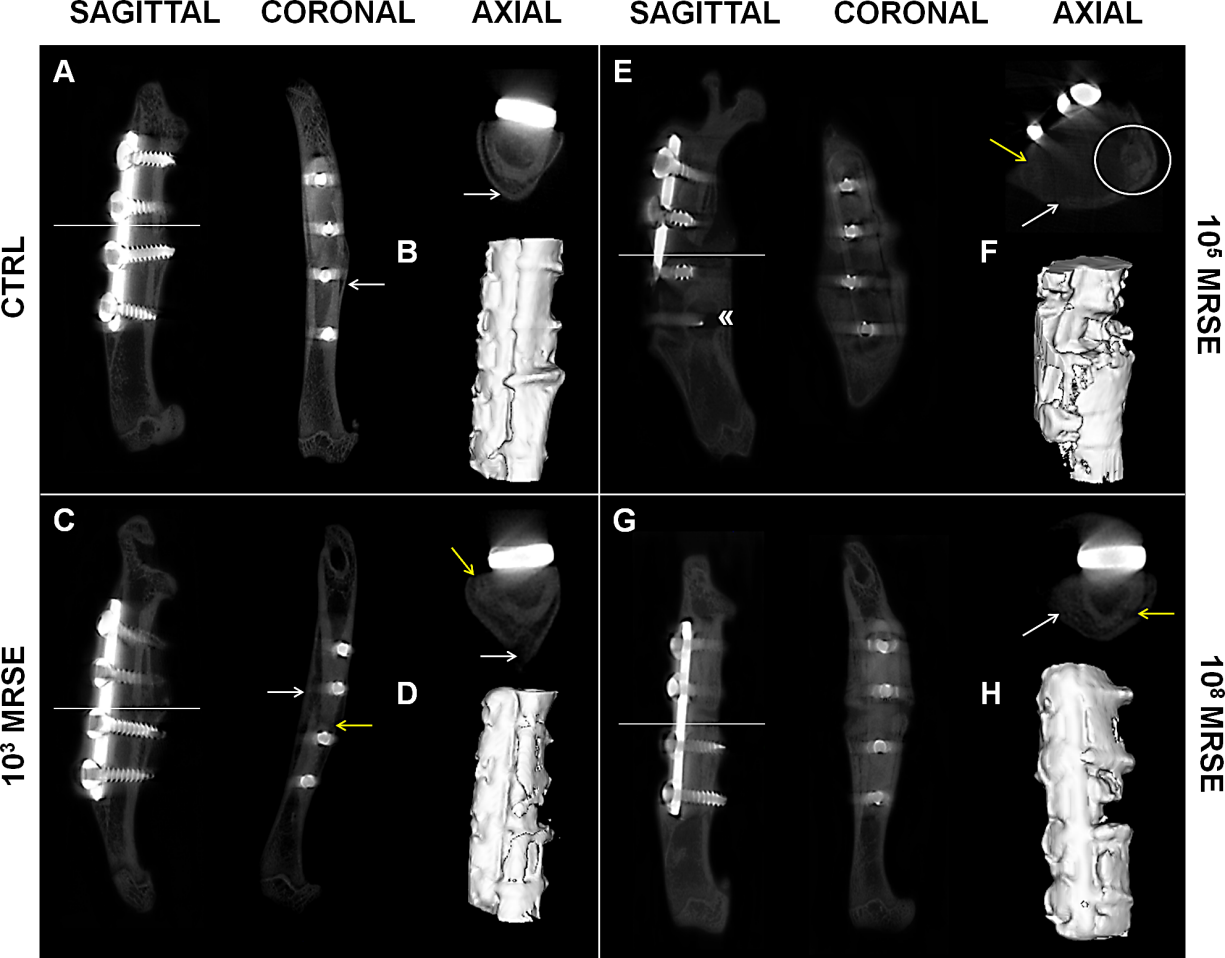
Qualitative micro-CT imaging and isosurface. The representative panel shows micro-CT images on the day of explantation. The sagittal, coronal and axial planes (A, C, E, G), as well as a three-dimensional isosurface reconstruction (B, D, F, H) were presented for the control (CTRL), and the 10^3^, 10^5^, and 10^8^ MRSE groups. Symbols indicate: cortical reaction (white arrows); loss of cortical wall and osteolysis (yellow arrows); peri-implant osteolysis («); presence of abscesses (white circle).

The micro-CT qualitative analysis showed no signs of osteomyelitis in the control group in all the analyzed planes (Fig 3A, sagittal, coronal, and axial). Some animals displayed a restricted cortical bone reaction, whereas a well-organized bone callus, remodeling, and a good bone encapsulation of the screws were present in all cases. One sample of the control group was not evaluated due to the mechanical loss of the proximal screws followed by a fracture dislocation because of a surgical inaccuracy. Thus, this subject was excluded from quantitative analyses and dedicated to SEM investigation. All valuable samples (100%) showed a fracture healing > 75% with mineralized cortices and bony bridging across the medullary canal (Fig 3B).

The 10^3^ MRSE group displayed variable response to bacterial infection. The 67% of samples showed signs of altered bone healing. In particular, they exhibited both a diffuse cortical bone thickening associated with focal loss of the cortical wall and mild osteolysis around the screws near to the fracture site (Fig 3C, sagittal, coronal, and axial). The fracture healing was < 75% and displayed mainly fibrous non-union (Fig 3D).

The 10^5^ MRSE group presented extensive signs of osteomyelitis both near the fracture site and extending to the metaphysis regions associated with severe osteolysis around the screws and resorption of the cortex, with a quite complete disruption of the bone integrity (Fig 3E, sagittal, coronal, and axial). Moreover, most of the samples demonstrated the presence of subcortical abscesses (Fig 3E, axial). The 83% of the samples showed a fracture healing < 75%, with absence of bony bridging, frequently associated with deformity of the femoral diaphysis (Fig 3F). One animal showed no signs of osteomyelitis and a fracture healing around 75%.

The 10^8^ MRSE group showed extensive cortical thickening, severe periosteal reaction, loss of cortical wall and severe osteolysis around the screws near to the fracture site (Fig 3G, sagittal, coronal, and axial). All samples (100%) showed a fracture healing < 75%, and displayed non-union extended across the entire bone (Fig 3H).

Assessing the percentage of bony bridging, no significant difference was measured between the 10^3^ MRSE group and the control group or the10^5^ and 10^8^ MRSE groups, as well as between the 10^5^ and 10^8^ MRSE group. The control group significantly differed from the 10^5^ and 10^8^ MRSE groups for *P*<0.05 and *P*<0.01, respectively.

**Fig 4:**
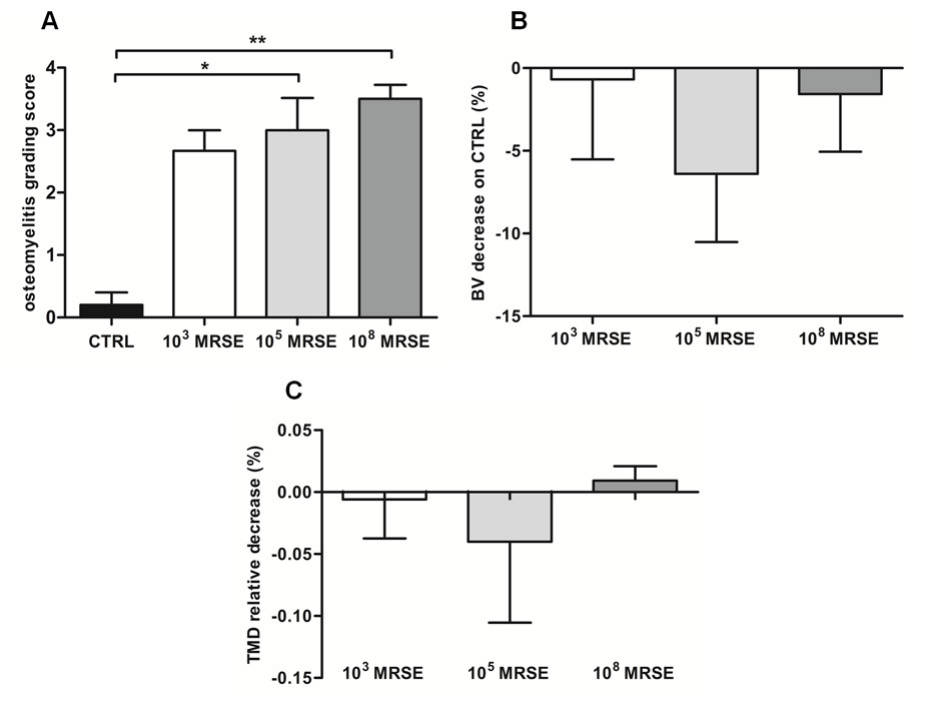
Micro-CT-based semiquantitative and quantitative analyses of bone structure. (A) Osteomyelitis grading score based on Odekerken’s scale. (B) Bone volume (BV) quantitative analysis of the infected groups normalized on the control group, reported as a percentage. (C) Tissue mineral density (TMD) quantitative analysis of the infected groups normalized on the control group, reported as a percentage. Comparisons among groups were analyzed by one-way ANOVA. Statistical significance for *P*<0.05 (*), n = 6.

The interrater reliability of blind scoring based on the Odekerken’s scale was excellent—ICC 0.83 (95% CI 0.51, 0.94)—and highlighted a significant higher osteomyelitis grade in the 10^5^ and 10^8^ MRSE groups, but not for the 10^3^ MRSE group, compared to the controls (*P*<0.05) (Fig 4A). The quantifications of BV and TMD were reported as percentage of decrease with respect to the control group in which, despite no significant difference was found, a lower BV was measured for all the infected groups with a higher trend in the 10^5^ MRSE group (Fig 4B) as well as for the TMD with the exception of the 10^8^ MRSE group (Fig 4C).

### Microbiological tests

No bacterial growth was observed in the control group (non-detectable values, n.d., ≤ L.o.D.). There was not a significant difference between the control group (n.d.) and the 10^3^ MRSE group (1.5±1 CFU/g explant, mean ± standard deviation), in which bacteria were found in three animals out of five. Considerable bacterial counts were found in samples of the 10^5^ and 10^8^ MRSE groups (10.33±9.5 and 20.33±27.4 CFU/g explant, respectively) with a significant difference compared to the control group (*P*<0.05 and *P*<0.01, respectively), as well as between the 10^3^ and 10^8^ MRSE groups a difference was measured (*P*<0.05). No significant differences in DNA sequences between strains isolated from animal explants and the parental strain were observed. In particular, all the isolated bacteria from rat explants were susceptible to gentamicin, vancomycin and fusidic acid (MIC ≤ 0.5μg/ml), erythromycin and daptomycin (MIC ≤ 0.25μg/ml), clindamycin and tigecycline (MIC ≤ 0.12μg/ml), linezolid and tetracycline (MIC ≤ 1μg/ml), teicoplanin (MIC = 4μg/ml), trimethoprim/sulfamethoxazole (MIC ≤ 10μg/ml) while they were resistant to benzylpenicillin (MIC ≥ 0.5μg/ml), oxacillin, cefazolin, rifampicin and levofloxacin (MIC ≥ 4μg/ml). As far as biofilm production was concerned, all the isolated bacteria were classified as strong biofilm producers as well as the parental strain, without significant differences among 10^3^, 10^5^ and 10^8^ CFU inoculum groups, as reported in Table 1.

**Table 1:** Biofilm production of *S*. *epidermidis* isolated from the infected rats (ODs vs ODc ratio).

GROUPS	EXPLANTS				
	A	B	C	D	E
**10**^**3**^ **MRSE**	6.26	6.37	n.d.	6.47	n.d.
**10**^**5**^ **MRSE**	6.28	6.58	5.67	6.54	6.14
**10**^**8**^ **MRSE**	6.53	6.48	5.78	6.28	6.21

n.d. = non-detectable

### Histological analysis

**Fig 5:**
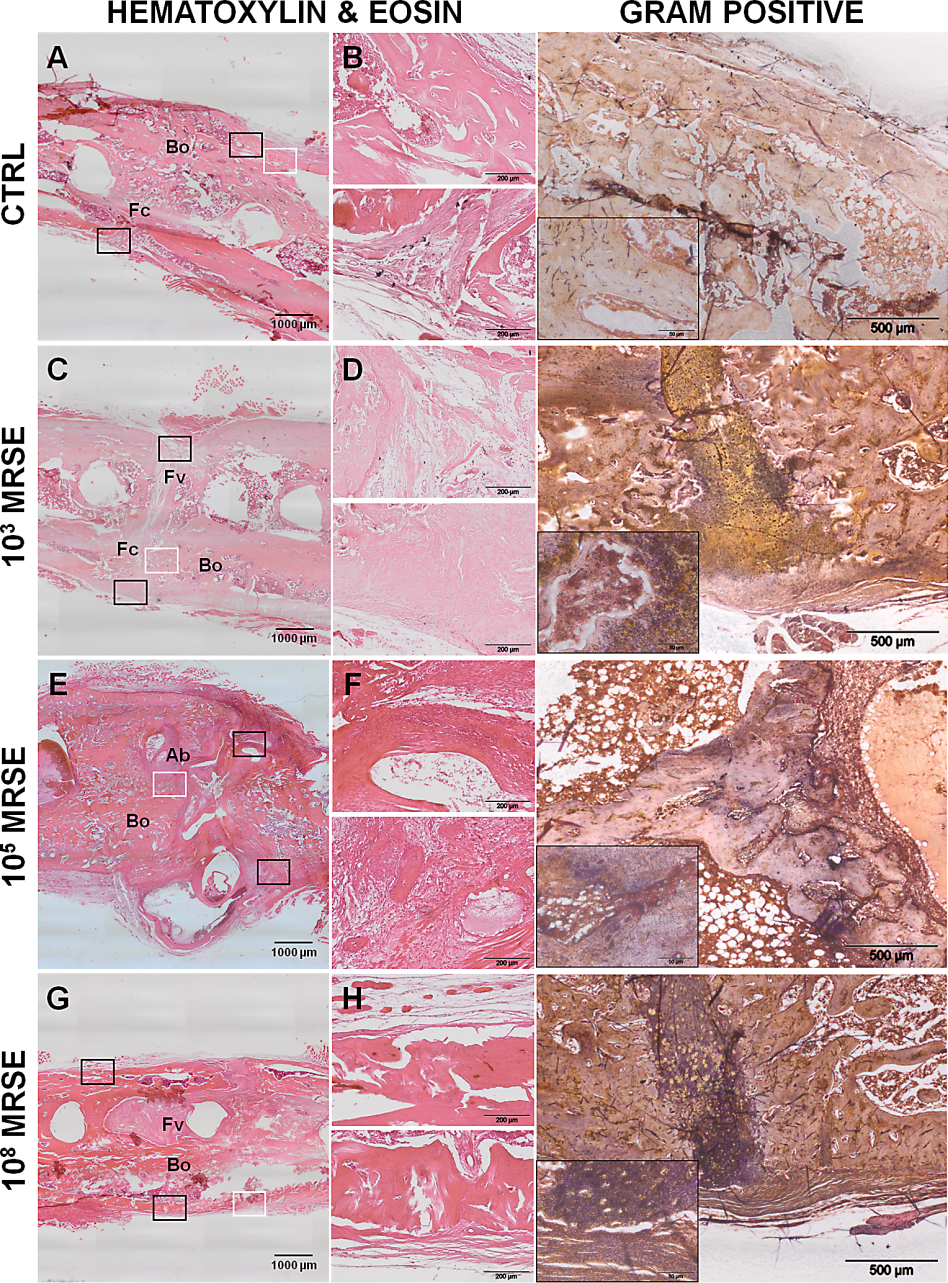
Histological analysis at the day of explantation. The representative panel shows H&E and Gram staining in the four groups. The panels depict an overview of the samples, magnification 2x, scale bar 1000μm (A, C, E, G). The panels depict areas of interest of the black boxes, magnification 10x, scale bar 200μm (B, D, F, H). The Gram staining depicts bacterial colonies identified by the white boxes, magnification 4x, scale bar 500μm and specific regions within the small boxes, magnification 40x, scale bar 50μm. Bo, bone; Fc, fibrocartilaginous callus; Fv, fibrovascular tissue; Ab, abscesses.

The histological analysis confirmed the results obtained by micro-CT scans in terms of percentage of fracture healing and bone structure in the control group (Fig 5A). Specifically, fractures appeared closed with a great amount of new bone formation in a remodeling phase, sometimes associated with a mild cortical thickening (woven bone) (Fig 5B, upper box). The small portion of unclosed fracture displayed a well-organized fibrocartilaginous tissue (Fig 5B, lower box). No signs of osteomyelitis were detected as also displayed by the absence of bacteria reported by the Gram-positive staining.

As aforementioned, animals of the 10^3^ MRSE group showed a variable response to bacterial infection. Animals with a microbiological detectable infection showed an incomplete bone healing (Fig 5C) characterized by a great formation of fibrovascular tissue disseminated with scarce inflammatory cells (Fig 5D, upper box) and only sporadically replaced by cartilaginous tissue (Fig 5D, lower box). The cortices appeared uniformly enlarged with few areas of bone remodeling. Gram staining detected areas disseminated with Gram-positive cocci. Differently, infected rats, in which no bacteria were microbiologically retrieved, showed histological features resembling the findings of the control group.

The 10^5^ MRSE group showed a complete disorganization of the bone structure, the missing of cortical bridging with non-union establishment (Fig 5E). Severe signs of osteomyelitis were found: extensive periosteal reaction, cortical thickening, myeloid hyperplasia, a diffuse presence of polymorphonuclear cells in the granulation tissue (Fig 5F, upper box), multiple subperiosteal, intracortical and medullary abscesses and hematomas, and bone sequestra (Fig 5F, lower box).

The severe osteomyelitis was also sustained by the presence of gram-positive bacteria in the bone and granulation tissue.

The histology of the 10^8^ MRSE group confirmed the results obtained by micro-CT scans. The analysis depicted a great deposition of fibrovascular tissue permeating from the cortical margin to the medullary canal, surrounding screws and embedding numerous polymorphonuclear cells (Fig 5G). The severe osteomyelitis led to a massive cortical and endosteal osteolysis (Fig 5H, upper box). Subperiosteal sequestra were found together with a moderate periosteal reaction (Fig 5H, lower box). Gram staining identified a massive presence of bacteria both intraosseous and within the periosteal and subperiosteal area.

**Fig 6:**
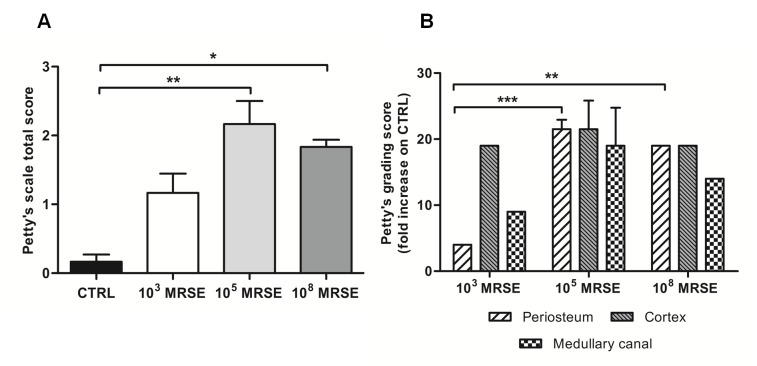
Histological score based on Petty’s scale. The histogram compares the semiquantitative score performed on the periosteum, cortex and medullary canal regarding the osteomyelitis signs. (A) Total score analysis. (B) Fold increase of the grading scale of the infected groups with respect to the control group. Comparisons among groups were analyzed by one-way ANOVA. Statistical significance for *P*<0.05 (*), *P*<0.01 (**), *P*<0.001 (***), n = 5.

The interrater reliability of blind scoring based on the Petty’s scale was excellent—ICC 0.82 (95% CI 0.62, 0.92). The histogram in Fig 6A depicts the total score, in which the control group showed no signs of bone infection and the 10^3^ MRSE group displayed only a mild inflammatory reaction with particular reference to the cortex, as shown in Fig 6B. A moderate to severe osteomyelitis was found in the 10^5^ and 10^8^ MRSE groups with a significant difference with respect to the control group for *P*<0.01 and 0.05, respectively. Moreover, a significant difference was found in terms of periosteal reaction between the 10^3^ MRSE group and both the 10^5^ and 10^8^ MRSE groups for *P*<0.001 and *P*<0.01, respectively.

### Scanning electron microscopy analysis

**Fig 7:**
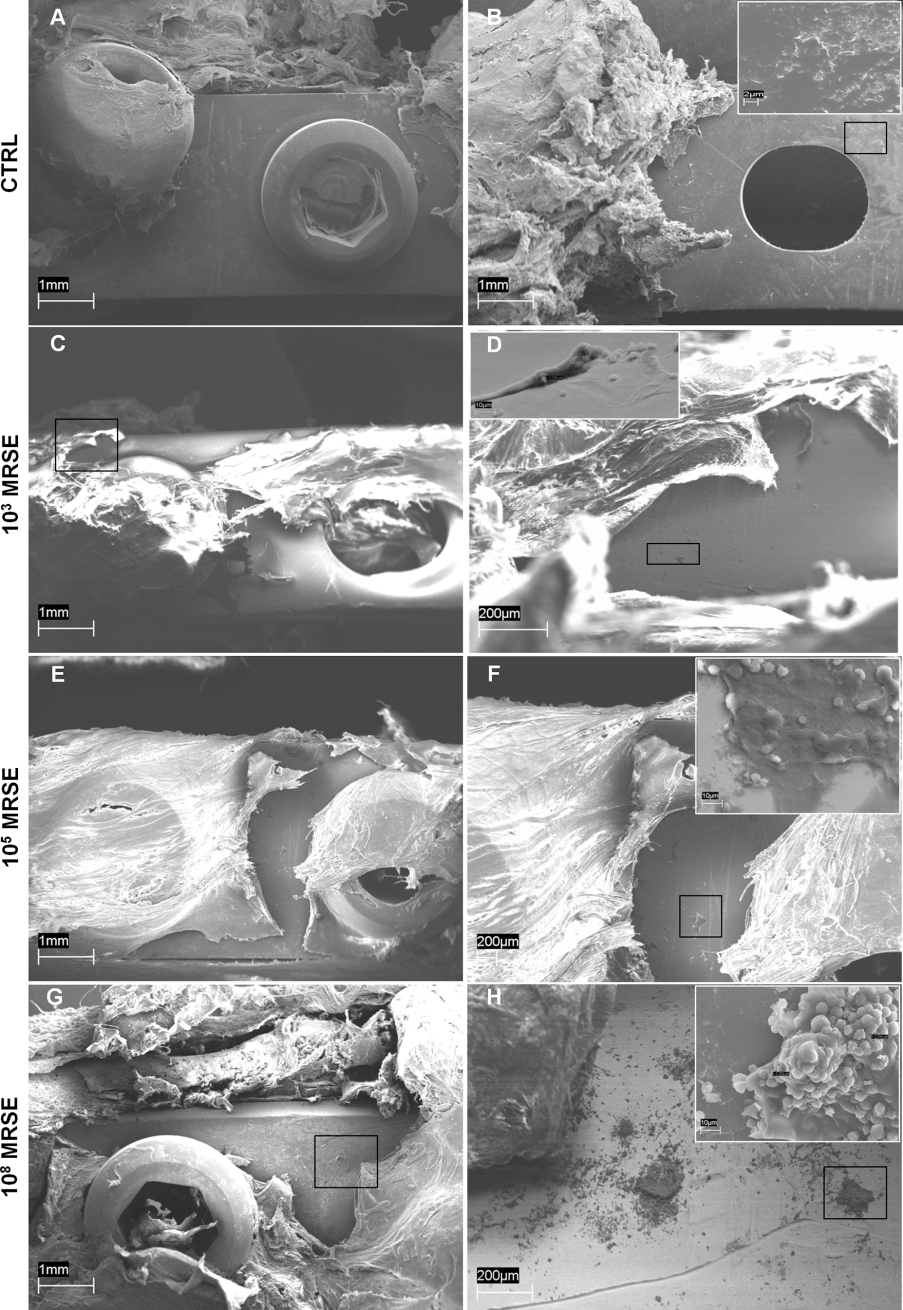
Representative photographs of SEM analysis of biofilm formation. Absence of biofilm formation in the control group either on the top (A) or on the bottom (B) of the implanted plate (magnification 40x, scale bar 1mm) with the presence of bony bridging structure on the bone-implant interface (B small box, magnification 6700x, scale bar 2μm). Presence of few coccidal bacteria on the top of the plate (C, D, magnifications 40x and 250x, scale bars 1mm and 200μm, respectively) occasionally coated with mucoid material in the 10^3^MRSE group (D small box, magnification 3000x scale bar 10μm). Presence of cocci within the peri-implant fibrous tissues covering the plate and on the top of the plate (E, magnification 40x, scale bar 1mm), free or partially embedded into mucous-gelatinous matrix in the 10^5^MRSE group (F, magnification 70x, scale bar 200μm; F small box, magnification 3000x scale bar 10μm). Clusters of cocci adhering to the top of the plate surface (G, H, magnifications 40x and 90x, scale bars 1mm and 200μm, respectively) completely embedded in a well-organized biofilm matrix in the 10^8^MRSE group (H small box, magnification 3000x, scale bar 10μm).

The control group without *S*. *epidermidis* contamination was completely free from biofilm formation on the surface of the implanted stainless steel plate both on its top (Fig 7A) and bottom side (Fig 7B), where the presence of bony bridging on the bone-implant interface was detected (Fig 7B, small box). A little presence of free cocci, ranging from 1–2 μm of diameter, was detected on the plate surface in the 10^3^ MRSE group, in which a scarce mucoid material occasionally coated cocci (Fig 7C and 7D). Differently, in the 10^5^ MRSE group, bacteria were mainly present on the plate surface and within the peri-implantar fibrous tissues covering the plate, free or partially embedded into mucous-gelatinous matrix (Fig 7E and 7F). As expected, in the 10^8^ MRSE group, several clusters of cocci were identified adherent at the top and bottom of the plate surface (Fig 7G and 7H) completely embedded in a well-organized and three-dimensional self-produced extracellular polymer structure, the biofilm, able to entrap also platelets, erythrocytes and polymorphonucleated cells (Fig 7H, small box).

## Discussion

Postoperative infections and invasive trauma causing septic non-union are still a major clinical problem that requires specific preclinical models. An interesting model has been proposed by Chen and colleagues [17] combining a critical bone defect in the rat femur with a local injection of *S*. *aureus*. This model may not effectively characterize the clinical condition in which septic non-unions develop. Actually, critical bone defects do not heal spontaneously, thus *in vivo* non-union models should avoid the combination of critical defect and bacterial inoculation to better understand the pathogenesis of infection in fractures, as also suggested by others [32, 33]. In our study, we proposed a standardized osteotomy model devoid of a critical bone defect associated with a local infection. This approach aims to better understand the role of bacteria in the non-union development with particular reference to subclinical contaminations and to generate a useful preclinical model of non-union-related infection for future therapeutic approaches. Similarly, Alt and colleagues [1] established a model of *S*. *aureus* non-unions of the tibia after intramedullary fixation. To resemble human procedures for complicated femoral fractures, we provided a direct fracture fixation with plates, we used a pathogen commonly involved in implant colonization and we administered routine antimicrobial prophylaxis. *S*. *epidermidis* biofilm formation is a well-known virulence factor in the development of implant infection [34]. Thus, to better recreate a human clinical setting, we developed a model taking advantage of a prosthetic-derived MRSE-strain selected for its ability to produce biofilm. The antibiotic susceptibility of the selected MRSE-strain was comparable to that of collection numbered ATCC35984, particularly referred to daptomycin and vancomycin [18]. Despite similar antibiotic susceptibility to ATCC35984, MIC values for vancomycin and daptomycin of our clinical strain were lower than the referenced one [18].

Moreover, we treated animals with a preoperative systemic, broad-spectrum cephalosporin (cefazolin), since it is considered the first choice in orthopaedic prophylaxis according to the specific guidelines [35]. Thus, we tried to prevent other than MRSE contaminations by using cefazolin to have a deeper insight in this specific bacterial infection. Again, male animals were selected to avoid the influence of hormonal cycles on bone repair and turnover, as also suggested by others [33]. In the literature scenario, there are several studies on osteomyelitis or septic arthritis animal models, but few studies report models of infections during the fracture healing by employing *S*. *aureus* as main strain [1,16, 17, 36–38]. Differently, we proposed a dose-dependent *S*. *epidermidis*-induced non-union model, belonging *S*. *epidermidis* to the normal skin flora and being responsible for most of the metal implant infections [13, 39]. To our best knowledge, there are not currently animal models simulating a *S*. *epidermidis*-related non-union of the femur after internal plate stabilization. In fact, only few studies describe the MRSE-related orthopaedic infections mainly focused on prosthetic joint infection [22] or intramedullary osteomyelitis [24].

In our results, the changes in b.w. and neutrophil count were correlated with the host response to infection. The b.w. loss reflected the dose-dependent trend in the infected rats, as well as the increase of the neutrophils during the acute phase of infection. Overall, most of the analyses highlighted a worse condition in the 10^5^ MRSE group compared to the others. The recovery of the 10^8^ MRSE group in terms of both b.w. and the systemic neutrophil count could be related to the capability of the formed biofilm to locally attract activated neutrophils reducing their presence in the circulating blood. This finding was histologically supported by the abundant presence of local polymorphonuclear cells and severe host tissue damages. Hence, this suggested a quicker, stronger and mature biofilm formation induced by the higher dose of injected bacteria (10^8^ MRSE) with respect to the other infected groups, as demonstrated by Gram staining, microbiological and SEM analyses. Thus, the greater amount of biofilm with a high mechanical stability allowed bacteria to escape from neutrophil assault. This supported the low susceptibility to phagocytic destruction by neutrophils of the *S*. *epidermidis* [40]. The inability to kill bacteria associated with a great amount of protective biofilm slowed the cocci metabolic rate leading to a chronic osteomyelitis development and eventually delaying the fracture bone healing [13]. *S*. *epidermidis* autolysin (AltE) has been suggested to mediate the primary attachment to the implant surface and to bind vitronectin, an abundant glycoprotein found in serum, extracellular matrix and bone, promoting cocci spreading [41]. The autolysin/adhesin system binds fibrinogen, fibronectin, and vitronectin in a bacterial dose-dependent fashion [41], explaining the greater amount of biofilm formation in the 10^8^ MRSE group compared to the 10^5^ MRSE one. Moreover, *S*. *epidermidis* embedded in biofilm commonly produces quorum-sensing controlled virulence factors as the one encoded by the accessory gene regulator *(agr)* system. In addition, the transcription of *Agr* gene is also induced by the presence of neutrophils and regulates biofilm formation in *S*. *epidermidis* by acting on AtlE and toxin expression that cause a rapid lysis of local polymorphonuclear cells [10, 42]. Therefore, the vicious circle of neutrophils promoted the biofilm formation and aggravated the local inflammatory response. Although *S*. *epidermidis* has a lower pathogenic potential, most staphylococcal infections are chronic especially in patients with associated predisposing factors such as diabetes or peripheral vascular disease, as we have previously demonstrated [31].

Differently, the 10^5^ MRSE group showed a more severe condition in terms of osteomyelitis signs and non-union establishment, as properly supported by micro-CT and histological analysis, despite a lower detection of bacterial growth. In this group, subacute osteomyelitis was characterized by abscesses at the site of bacterial colonization frequently associated with vascular and bone damages, like cortical resorption and sequestra. Sequestra represent foci of recurrent infection that could lead to the chronic osteomyelitis development over time. Accordingly, in these rats, the subacute infection was also related to a higher symptomatic status in terms of b.w. decrease and circulating neutrophils, as also supported by others [43]. Reducing the bacterial load and consequently the biofilm production in the 10^5^ MRSE respect to the 10^8^ MRSE group could be responsible of a higher presence of free cocci in the fracture site. In fact, this lower microbial dose induced a subacute host response fighting against cocci, with both maintenance of high levels of circulating neutrophils and local cellular immune reaction (abscess formation). This phenomenon could be reasonably controlled by using appropriate antimicrobial treatment for MRSE, considering that bacteria in such specific case are poorly embedded in a weak and immature extracellular matrix.

More interestingly, the 10^3^ MRSE group displayed an inconsistent grade of infection and fracture healing. This could be related to the specific host immune system that was able to spontaneously eradicate the infection determined by a low-grade of MRSE injection in 33% of rats, as demonstrated also by other authors [28]. According to our experience, this group showed subclinical features of infected fractures, in which the bone repair or damages are filtered by the host immune defense and bacteria did not trigger signs or symptoms of a post-surgical infection. Findings of our study, in which a low grade of *S*. *epidermidis* administration in the presence of metal implants seems to be a good model for subclinical perioperative infections, are consistent with those supported by Qin and colleagues in an implant-related osteomyelitis in a rat model [24]. The spontaneous eradication of infection occurred in some cases of the 10^3^ MRSE group suggests the inadequacy of low grade infection in creating a reliable and reproducible model of infected non-unions, supporting another study on *S*. *epidermidis* graft infections [44]. Conversely, it may represent a useful approach to have a deeper insight of the subclinical event in terms of pathogens and host response.

The control group was free of clinical and diagnostic signs of infection and showed a quite complete fracture healing, as expected. The restrict cortical bone reaction detected by micro-CT is due to the periosteal elevation performed during surgery to properly create the fracture by means of a circular saw. According to some authors, the cautery-induced periosteal damage induced an atrophic nonunion [45]. In our study, we carried out a simple periosteal cut and elevation without provoking necrotic damages. This variability was adopted in all animal groups.

In the development of our models, some limitations were encountered in the micro-CT imaging related to the scattering artifacts of the stainless steel plates and screws. This phenomenon was particularly limiting for the quantitative analyses. To overcome this limit, the use of titanium implants could be helpful to reduce scattering artifacts and to apply validated protocols for micro-CT acquisition. Anyway, thanks to the combination of several diagnostic techniques, our bone analysis provided adequate information on fracture healing and osteomyelitis associated to microbial infections. Our modeling is reliable for the assessment of osteomyelitis and fracture healing, offering a good correlation of their typical features. Moreover, the small number of animals included in this research could be also considered a limitation, despite offering an innovative and interesting preclinical model. Indeed, at this writing, there are not available rodent models describing the use of *S*. *epidermidis* to determine an orthopaedic infection associated with non-union fractures. This is also the first time that the impact of bacterial load has been investigated with regard to bone healing after fracture and osteosynthesis and that the effect on bone healing of a subclinical infection due to *S*. *epidermidis* contamination of a metallic plate is shown. Although this research is a pilot study, the model appears both as a more traditional tool, designed to study implant-related infections due to a common pathogen, like *S*. *epidermidis*, and as a more innovative way to investigate the underestimated effect of subclinical contamination of bone due to trauma or surgery.

## Conclusions

Our models of subclinical and evident orthopaedic infection constitute clinically relevant tools for studying prophylactic and therapeutic strategies or biofilm pathogenesis in infected non-union establishment as well as models for the assessment of sophisticated diagnostic approaches. Importantly, our modeling could be used in the next feature to characterize by proteomics the adaptation and changes of bacteria during the different stages of the infective process and biofilm formation.

## Abbreviations

b.w.: Body weight
BV: bone volume
CI: confidential interval
CTRL: control group
DTT: dithiothreitol
L.o.D.: limit of detection
MRSE: methicillin-resistant *S*. *epidermidis*
OD: optical density
RT: room temperature
TMD: tissue mineral density
